# Risk factors for arterial versus venous thrombosis in polycythemia vera: a single center experience in 587 patients

**DOI:** 10.1038/s41408-017-0035-6

**Published:** 2017-12-27

**Authors:** S. Cerquozzi, D. Barraco, T. Lasho, C. Finke, C. A. Hanson, R. P. Ketterling, A. Pardanani, N. Gangat, A. Tefferi

**Affiliations:** 1 Division of Hematology, Department of Internal Medicine, University of Calgary, Calgary, AB Canada; 2 Division of Hematology, Department of Internal Medicine, Mayo Clinic, Rochester, MN USA; 3 Division of Hematopathology, Department of Laboratory Medicine and Pathology, Mayo Clinic, Rochester, MN USA

## Abstract

In a recent International Working Group on Myeloproliferative Neoplasms Research and Treatment (IWG-MRT) study, prior arterial events and hypertension were predictors of subsequent arterial thrombosis whereas prior venous events and age ≥65 years predicted venous thrombosis in polycythemia vera (PV). In the current study, we sought to validate the above findings and identify additional predictors of arterial versus venous thrombosis. At a median follow up of 109 months, thrombosis after diagnosis occurred in 128 (22%) patients; 82 (14%) arterial and 57 (10%) venous events. On multivariate analysis, prior arterial events (<0.0001), hyperlipidemia (*p* = 0.03), and hypertension (*p* = 0.02) predicted subsequent arterial events. In comparison, prior venous events (*p* = 0.05), leukocytosis ≥11 × 10^9^/L (*p* = 0.002), and major hemorrhage (*p* = 0.02) were predictors of subsequent venous events. Salient associations with arterial thrombosis included age ≥ 60 years, hypertension, diabetes, hyperlipidemia and normal karyotype whereas age ≤ 60 years, females, palpable splenomegaly and history of major hemorrhage were associated with venous thrombosis. *TET2* or *ASXL1* mutations did not impact arterial nor venous thrombosis. In conclusion, we identify distinct associations for arterial versus venous thrombosis in PV and confirm that a prior arterial or venous thrombotic event is the most reliable predictor of subsequent events.

## Introduction

Polycythemia vera (PV) is a myeloproliferative neoplasm (MPN) characterized by clonal erythrocytosis resulting from constitutive activation of the Janus Kinase and Signal Transducer and Activator of Transcription (*JAK-STAT)* signal transduction pathway. The diagnosis of PV is based on the 2016 World Health Organization (WHO) criteria utilizing a composite assessment of clinical and laboratory features^1^. PV is associated with burdensome symptoms, reduced quality of life and thrombohemorrhagic complications with potential for myelofibrotic and/or leukemic transformation^2^. Thrombotic complications are a major cause of morbidity and mortality with a reported incidence of 12–39%^3^. Patients suffer from large vessel arterial and venous thrombosis, as well as microcirculatory symptoms such as dizziness, headaches, visual disturbances, erythromelalgia, distal paresthesia and acrocyanosis^3^. In the European Collaboration on Low-Dose Aspirin in Polycythemia Vera (ECLAP) prospective study, age older than 65 years and prior thrombotic events were identified as risk factors for cardiovascular events among PV patients^4^. Importantly, arterial and venous thrombosis are two biologically different processes with distinct risk factors. The International Working Group on Myeloproliferative Neoplasms Research and Treatment (IWG-MRT) identified prior arterial events and hypertension as risk factors for arterial thrombosis and prior venous events and age ≥ 65 years as risk factors for venous thrombosis in PV^5^. In this single center study, we sought to validate the findings of the IWG-MRT based on WHO 2016 PV diagnostic criteria, as well as identify additional risk factors for arterial versus venous thrombosis, including possible associations with the most frequent “non-driver” mutations: Tet methylcytosine dioxygenase 2 (*TET2)* (22%) and Additional sex combs like-1 (*ASXL1)* (12%)^6^.

## Material and methods

The current study was approved by the institutional review board of Mayo Clinic (Rochester, MN). Study patients were selected from our institutional database of MPN and fulfilled the 2016 WHO criteria for the diagnosis of PV^1^. Clinical and laboratory data was obtained from the time of diagnosis. Only major thrombotic events were considered specifically: 1) Arterial thrombosis: acute myocardial infarction (MI), angina pectoris, ischemic stroke, transient ischemic attack (TIA), peripheral arterial disease (PAD) or 2) Venous thrombosis: pulmonary embolism (PE), deep vein thrombosis (DVT), portal or mesenteric thrombosis (splanchnic vein thrombosis), cerebral sinus vein thrombosis. Thrombotic events were objectively identified based on diagnostic imaging. Post-PV thrombotic events were defined as events occurring ≥ 4 weeks following PV diagnosis. Major hemorrhage was defined based on International Society on Thrombosis and Hemostasis (ISTH) definitions as: gastrointestinal, internal organ, intraarticular, cerebrovascular, retroperitoneal bleed or any bleeding requiring medical and/or surgical intervention, hospitalization and/or resulting in death. Therapy in the study included low dose Aspirin (81 mg PO once daily) and cytoreductive therapy including: hydroxyurea, busulfan, interferon, anagrelide, and JAK inhibitors. Cytogenetic analysis and reporting was done according to the International System for Human Cytogenetic Nomenclature^7^. In addition to *JAK2V617F* and *exon 12* testing, mutation screening for *TET2* and *ASXL1* was performed according to conventional methods^6,8^. Differences in the distribution of continuous variables between categories were analyzed by either Mann–Whitney (for comparison of two groups) or Kruskal–Wallis test (comparison of three or more groups). Patient groups with nominal variables were compared by *χ* ^2^-test. Thrombosis-free survival (TFS) was determined from the time of diagnosis to the time of event occurrence after diagnosis (uncensored) or last contact/date of death (censored). All survival curves were prepared by the Kaplan–Meier methods and compared by the log-rank test. Cox proportional hazard regression model was applied to carry out multivariate analysis. We considered *p*-values less than 0.05 as significant. The Stat View (SAS Institute, Cary, NC, USA) statistical package was used for all calculations.

## Results

Among a total of 587 patients, the median age of our cohort was 60 years (range; 17–94 years) with 48% males. Based on current European Leukemia Net (ELN) classification^9^, 64% were “high risk” PV (age ≥ 60 years and/or history of prior thrombosis). Patient characteristics are outlined in Table 1. The incidence of cardiovascular risk factors was as follows: hypertension (42%), diabetes (9%), hyperlipidemia (21%), and history of active or remote smoking (29%). As expected, the majority of patients were *JAK2* mutated (99%) with 18 and 11% harboring *TET2* and *ASXL1* mutations, respectively. At a median follow up of 109 months, 14 and 4% of patients experienced myelofibrotic and leukemic transformation, respectively.

**Table 1:** Clinical and laboratory characteristics of 587 patients with PV

Variables	All patients (*n* = 587)
Age at diagnosis (years); median (range)	60 (17–94)
Age ≥ 60	289 (49%)
Females	303 (52%)
Leukocytes ≥ 11 × 10^9^/L (*N* = 505)	284 (56%)
Platelets, × 10^9^/L; median (range)	476 (41–2747)
Hypertension (*N* = 581)	242 (42%)
Diabetes (*N* = 584)	52 (9%)
Hyperlipidemia (*N* = 581)	123 (21%)
Active smokers (*N* = 575)	64 (11%)
Cytogenetics (*N* = 239)	
Normal karyotype	193 (81%)
Palpable splenomegaly (*N* = 506)	155 (31%)
Major hemorrhage (*N* = 519)	20 (4%)
Conventional PV risk stratification:	
Low risk	211 (36%)
High risk	376 (64%)
Treatments:	
Phlebotomy	472 (80%)
Low dose ASA	437 (74%)
Cytoreductive therapy	480 (82%)
*TET2* (*N* = 133)	24 (18%)
*ASXL1* (*N* = 133)	14 (11%)
Median follow-up	109 months
Leukemic transformation	23 (4%)
Myelofibrotic transformation	81 (14%)
Deaths	224 (28%)

*L* liter, *PV* polycythemia vera

A total of 235 (40%) patients experienced any thrombotic event which included 153 (26%) arterial and 104 (18%) venous thromboses. One hundred forty-six patients (25%) had a thrombotic event prior to or at the time of diagnosis followed by 40 (27%) of patients with a recurrent event after PV diagnosis. Overall, post diagnosis, a total of 128 (22%) thrombotic events occurred with 11 (5%) of PV patients having experienced both an arterial and venous event. Table 2 outlines the type of arterial and venous events with acute coronary syndrome (ACS) being the most common arterial events (45%) experienced before or at time of diagnosis and cerebrovascular events occurring most commonly post-diagnosis (44%). In comparison, splanchnic vein thrombosis (45%) represented the most common venous events before or at diagnosis while deep venous thrombosis (DVT) (44%) were more common venous events post-diagnosis.

**Table 2:** Details of thrombotic events experienced by patients before/at or after diagnosis of PV

**Thrombotic event**	**Thrombosis**	**Thrombosis**
	**Before or AT**	**After**
	**PV diagnosis**	**PV diagnosis**
Any thrombosis	146 patients (25%)	128 patients (22%)
Arterial thrombosis	110 (19%) events	82 (14%) events
ACS (MI and angina)	49 (45%)	30 (37%)
Cerebrovascular	45 (41%)	36 (44%)
Peripheral arterial disease	16 (15%)	16 (20%)
Venous thrombosis	69 (12%) events	57 (10%) events
DVT	25 (36%)	25 (44%)
PE	12 (17%)	10 (18%)
Splanchnic vein thrombosis	31 (45%)	22 (39%)
Cerebral sinus thrombosis	1 (1%)	0 (0%)

*ACS* acute coronary syndrome, *DVT* deep vein thrombosis, *PE* pulmonary embolism, *PV* polycythemia vera, *MI* myocardial infarction

**Fig. 1:**
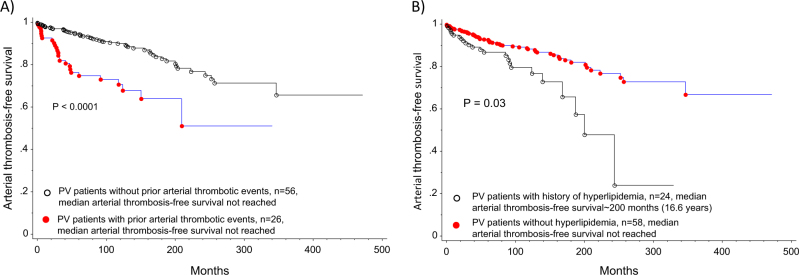
Arterial thrombosis-free survival based on multivariate analysis was significantly affected by **a** prior arterial events and **b** hyperlipidemia

**Fig. 2:**
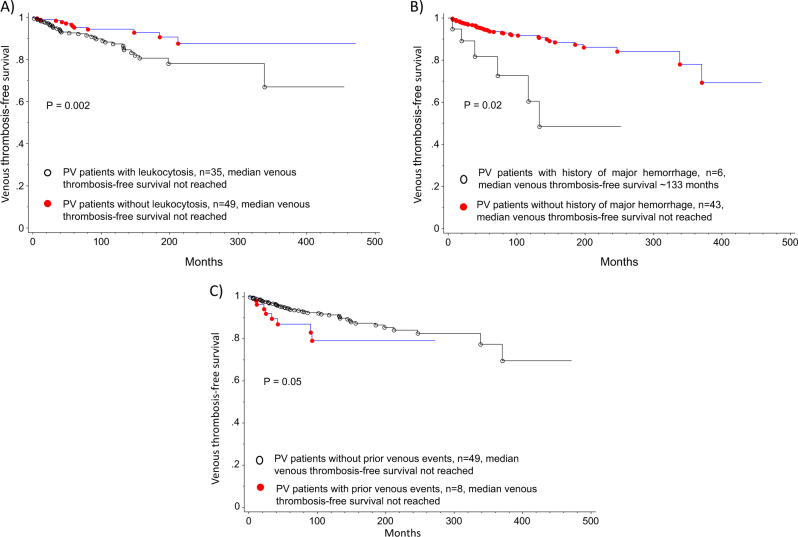
Venous thrombosis-free survival based on multivariate analysis was significantly affected by **a** leukocytosis, **b** major hemorrhage and **c** prior venous thrombosis

In multivariate analysis, prior thrombotic events (HR 1.9, CI: 1.2–2.9, *p* = 0.03) and leukocytosis (WBC ≥ 11 × 10^9^/L) (HR 1.3, CI: 0.9–2.0, *p* = 0.03) were predictive of subsequent thrombotic events in general. Specifically, prior arterial events (HR 2.7, CI: 1.7–4.5, *p* < 0.0001) and hyperlipidemia (HR 1.8, CI: 1.1–2.9, *p* = 0.03) were predictors of subsequent arterial events. In contrast, presence of major hemorrhage at diagnosis (HR 3.4, CI: 1.2–9.7, *p* = 0.02), leukocytosis (WBC ≥ 11 × 10^9^/L) (HR 2.0, CI: 1.1–3.8, *p* = 0.002) and prior venous events (HR 2.2, CI: 0.9–4.9, *p* = 0.05) were predictive of future venous events. Non-driver mutations (*TET2* and *ASXL1*) did not significantly influence neither arterial nor venous events. Table 3 summarizes results from both univariate and multivariate analysis for thrombosis-free survival. Based on significant parameters obtained on multivariate analysis, thrombosis-free survival data are shown in Figs. 1 and 2. Given the limited number of thrombotic events after diagnosis, we further explored clinical associations with arterial and venous thrombotic events occurring anytime either at or after PV diagnosis. As shown in Tables 4 and 5, the following salient associations were noted with arterial and venous events. Older patients (≥60years) (*p* = 0.006), patients with hypertension (*p* = 0.004), diabetes (*p* = 0.005), hyperlipidemia (*p* < 0.0001) or with normal cytogenetics (*p* = 0.01) experienced higher rates of arterial thrombosis. In contrast, younger patients (≤60 years) (*p* = 0.0005), females (*p* = 0.008), patients with palpable splenomegaly (*p* = 0.006) and a history of major hemorrhage (*p* = 0.0002) were more likely to experience venous thrombotic events. Patients who experienced a venous event were less likely to have cardiovascular risk factors: hypertension, hyperlipidemia or be active smokers.

**Table 3:** Univariate and Multivariate analysis of thrombosis-free survival among 587 patients with PV

**All thrombosis**	**Univariate analysis**	**Multivariate analysis**
Prior thrombotic events	HR 1.8, CI:1.2–2.6, *p* = 0.002	HR 1.9, CI: 1.2–2.9, *p* = 0.03
Leukocytosis (WBC ≥ 11 × 10^9^/L)	HR 1.4, CI: 0.9–2.1, *p* = 0.03	HR 1.3, CI: 0.9–2.0, *p* = 0.03
Arterial thrombosis		
Prior arterial events	HR 3.1, CI: 1.9–4.9, *p* < 0.0001	HR 2.7, CI:1.7–4.5, *p* < 0.0001
Hyperlipidemia	HR 2.2, CI:1.4–3.6, *p* = 0.001	HR 1.8, CI:1.1–2.9, *p* = 0.03
Hypertension	HR 1.7, CI: 1.1–2.6, *p* = 0.02	NS, *p* = 0.1
Venous thrombosis		
Prior venous events	HR 2.1, CI:1.0–4.7, *p* = 0.04	HR 2.2, CI: 0.9–4.9, *p* = 0.05
Leukocytosis (WBC ≥ 11 × 10^9^/L)	HR 2.1, CI: 1.1–3.9, *p* = 0.0009	HR 2.0, CI:1.1–3.8, *p* = 0.002
Major hemorrhage	HR 4.7, CI: 1.9–11.0, *p* = 0.0004	HR 3.4, CI:1.2–9.7, *p* = 0.02

*WBC* white blood count, *L* liter, *PV* polycythemia vera

**Table 4:** Clinical and laboratory features associated with arterial thrombosis among 587 patients with PV

**Variables**	**Patients without arterial thrombotic events**	**Patients with arterial thrombotic events**	***P*** **value**
	**(** ***n*** ** = 434)**	**(** ***n*** ** = 153)**	
Age at diagnosis (years), median (range)	59 (17–91)	63 (28–94)	0.001
Age ≥ 60, *n* (%)	199 (46%)	90 (59%)	0.006
Female, *n* (%)	227 (52%)	76 (50%)	0.6
Leukocytes, × 10^9^/L, median (range)	11.5 (3.8–59.3)	12.1 (4.3–171.6)	0.2
Leukocytes ≥ 11 × 10^9^/L, median (range) (*N* = 505)	207 (55%)	77 (58%)	0.6
Platelets, × 10^9^/L, median (range)	467 (44–2747)	494 (44–2747)	0.9
Palpable splenomegaly (*N* = 506)	125 (34%)	30 (26%)	0.02
Hypertension (*N* = 581)	164 (38%)	78 (52%)	0.004
Diabetes (*N* = 584)	30 (7%)	22 (14%)	0.005
Hyperlipidemia (*N* = 581)	70 (16%)	53 (35%)	<0.0001
Active smokers (*N* = 575)	51 (12%)	13 (9%)	0.3
Major hemorrhage (*N* = 519)	15 (4%)	5 (4%)	0.8
Cytogenetics (*N* = 239)			
Normal karyotype	162 (78%)	31 (97%)	0.01
*JAK2* (*N* = 560)	406 (99%)	146 (97%)	0.1
*TET2* (*N* = 133)	18 (20%)	6 (14%)	0.4
*ASXL1* (*N* = 133)	9 (10%)	5 (12%)	0.8

*L* liter, *PV* polycythemia vera

**Table 5:** Clinical and laboratory features associated with venous thrombosis among 587 patients with PV

**Variables**	**Patients without venous thrombotic events**	**Patients with venous thrombotic events**	***P*** **value**
	**(** ***n*** ** = 483)**	**(** ***n*** ** = 104)**	
Age at diagnosis (years), median (range)	61 (17–94)	54 (17–89)	<0.0001
Age ≥ 60 years, *n* (%)	254 (53%)	35 (34%)	0.0005
Female, *n* (%)	237 (49%)	66 (63%)	0.008
Leukocytes, × 10^9^/L, median (range)	11.5 (3.8–59.3)	12.3 (4.7–171.6)	0.5
Leukocytes ≥ 11 × 10^9^/L, median (range) (*N* = 505)	230 (55%)	54 (61%)	0.4
Platelets, × 10^9^/L, median (range)	493 (41–2747)	408 (97–1550)	0.001
Palpable splenomegaly (*N* = 506)	118 (28%)	37 (43%)	0.006
Erythromelagia (*N* = 504)	28 (7%)	10 (11%)	0.2
Hypertension (*N* = 581)	211 (44%)	31 (30%)	0.009
Diabetes (*N* = 584)	43 (9%)	9 (9%)	0.9
Hyperlipidemia (*N* = 581)	112 (23%)	11 (11%)	0.004
Active smokers (*N* = 575)	59 (12%)	5 (5%)	0.03
Major hemorrhage, (*N* = 519)	10 (2%)	10 (11%)	0.0002
Cytogenetic (*N* = 239)			
Normal karyotype	178 (80%)	15 (88%)	0.4
*JAK2* (*N* = 560)	452 (98%)	100 (99%)	0.7
*TET2 (N* = 133)	20 (19%)	4 (15%)	0.7
*ASXL1 (N* = 133)	11 (10%)	3 (12%)	0.9

*L* liter, *PV* polycythemia vera

## Discussion

In the current large series of PV patients that were evaluated and carefully followed at a single institution, we were able to systematically analyze the differences in risk factors for arterial versus venous thrombosis. In our study, the incidence of arterial (17%) and venous thrombosis (9%) before or at PV diagnosis, were similar to prior studies; ECLAP (27 and 11%, respectively), CYTO-PV (17 and 12%, respectively) and IWG-MRT study (16 and 7.4%, respectively)^4,5,10,^. Twenty-two percent of our patients experienced a thrombotic event after diagnosis (2.4%/year), which was also similar to the CYTO-PV and ECLAP studies (2.7/year and 2.6%/year, respectively), but lower than the IWG-MRT study (4.4%/year)^4,5,10^.

We confirm that a history of arterial and/or venous thrombosis is the most reliable risk factor for future arterial and venous events as shown in the IWG-MRT study^5^. A novel observation is the association of leukocytosis with increased risk of all thrombotic events, specifically venous thrombosis which differs from the findings of the ECLAP study in which leukocytosis ≥ 15 × 10^9^/L was associated with increased cardiovascular events among PV patients^4^. Leukocytosis is also a well-established risk factor for thrombosis and inferior survival among essential thrombocytosis (ET) patients^11–13^.

The role of cardiovascular risk factors in thrombosis was explored, with hyperlipidemia having an impact on future arterial thrombosis. As expected, we identified older age (≥ 60 years), hypertension, diabetes, hyperlipidemia, and normal karyotype to be associated with increased risk of overall arterial thrombosis. In contrast, younger patients, females, patients with palpable splenomegaly and a history of major hemorrhage were more likely to experience a venous thrombotic event. The IWG-MRT had also identified this association of female gender and venous thrombosis which may be related to use of concomitant hormonal therapy but was not specifically evaluated in either study^5^. The association of splenomegaly and thrombosis has been evaluated in ET with conflicting results^14,15^.

In conclusion, our study reinforces the importance of a history of thrombosis in predicting future thrombotic events and supports that arterial and venous events are distinct entities with specific risk factors that require careful evaluation and management. Additionally, the non-driver *TET2* and *ASXL1* mutations did not impact arterial nor venous thrombosis. Further studies are needed to conclusively confirm the role of specific risk factors identified for arterial versus venous thrombosis.
